# Male attitudes towards family planning in the Limpopo province of South Africa

**DOI:** 10.4102/safp.v64i1.5587

**Published:** 2022-10-06

**Authors:** Ndifelani D. Radzuma, Gert J.O. Marincowitz, Clara Marincowitz

**Affiliations:** 1 Department of Family Medicine, Faculty of Health Sciences, University of Limpopo, Mankweng, South Africa; 2 Limpopo Health, Kgapane Hospital, Kgapane, South Africa; 3 Department of Family Medicine, Limpopo Department of Health, Mankweng Hospital, Mankweng, South Africa; 4 Department of Psychiatry, Faculty of Health Sciences, University of Stellenbosch, Tygerberg, South Africa; 5 SA Medical Research Council, Cape Town, South Africa

**Keywords:** family planning, contraception, rural men, perceptions, attitudes, decision-making, communication, focus group discussion

## Abstract

**Background:**

Women often do not receive support from their partners with regards to family planning (FP), which can lead to hesitancy and inconsistent use. This study sought to understand the male attitudes that contribute to this.

**Methods:**

A qualitative descriptive study was conducted in 2019 using focus group discussions (FGDs) with purposively selected men aged ≥ 25 years and in a relationship with a woman of childbearing age. An open-ended question guide was used to explore men’s perceptions regarding FP. The discussions were recorded, translated and transcribed verbatim, whereafter transcripts were coded and analysed thematically.

**Results:**

Three major themes were identified, namely: (1) the advantages of FP, including financial benefits and the prevention of sexually transmitted infections and unwanted pregnancy; (2) the disadvantages of FP, including perceived adverse effects on men and women, as well as marital difficulties; and (3) the exclusion of men from FP by health workers and their partners.

**Conclusion:**

Men felt ambivalent towards FP. They were aware of the benefits thereof, but were hesitant to allow their female partners to use contraceptives, because of several misconceptions about the adverse effects. This underscores the need to involve men in FP programmes.

## Introduction

In rural South Africa, many women are forced to carry the burden of preventing unwanted pregnancies alone. They often do not receive support from their partners with regards to family planning (FP), and additionally, some women are hesitant to use contraceptives because of their male partners’ resistance and fear of consequent spousal retaliation.

Several factors have been shown to contribute to the low uptake or inconsistent use of FP. These include refusal by husbands or partners, myths regarding FP, as well as cultural issues.^1^ Unintended pregnancies are a problem that affect not only women but also their families and society as a whole. Despite the availability of services, unintended pregnancies are still a problem in South Africa and worldwide.

Currently, South Africa is experiencing key reproductive health challenges that are embedded in sociopolitical variables.^1^ Most women in rural areas rely on men for the provision of all their basic daily needs. As a result, this may affect their health when it comes to birth control, since they feel that they cannot make decisions regarding the issue independently. Unmet needs for FP remain a worldwide challenge, and in 2018, approximately 218 million women in developing countries could not access and utilise FP.^2^ Since 1970, there has been a global expansion in contraceptive prevalence, as well as a reduction in unmet need; however, sub-Saharan Africa has continued to lag in this regard. This region has the lowest contraceptive prevalence, at a rate of 24%, and a significant level of unmet need (25%).^3^

While FP services have traditionally targeted women, there is growing recognition that reproductive health is the joint responsibility of both women and men.^4^ Interest in involving men in FP is increasing, not only from a female reproductive health perspective but also to address men’s own health concerns, as well as to achieve the Sustainable Development Goal of reducing maternal mortality and human immunodeficiency virus (HIV) transmission.

Growing evidence suggests that male involvement in FP may increase women’s contraceptive uptake.^5,6^ This is likely a result of the fact that, particularly in rural areas, men remain the dominant decision-makers. It is therefore important to understand male attitudes towards contraceptive practices, especially since involving men in FP programmes has been shown to reduce their opposition and consequently spike birth control uptake and continuation.^5,6^ This has far-reaching effects with a reduction in the number of unintended pregnancies, an improvement in the socio-economic status of families and a decrease in pregnancy-related risks and infant mortality.

The first step towards involving men in FP programmes is to understand their knowledge and attitudes toward contraceptives. This is what the following study aimed to achieve by using focus group discussions (FGDs) to investigate male attitudes towards the birth control measures practised by their partners in the Limpopo province of South Africa.

## Methodology

A qualitative descriptive study was conducted, collecting data from two FGDs. The participants were selected purposively with the following inclusion criteria: male, aged ≥ 25 years and in a intimate relationship with a woman of childbearing age. Healthcare workers and mental healthcare users were excluded. Community healthcare workers and data capturers from two clinics recruited the participants through face-to-face interaction either in their homes or at the clinics. Additionally those selected were requested to refer other potential participants.

Thirty-one participants were suitable for inclusion; however, only 26 men took part in the two FGDs, which were conducted on a weekend in August 2019 to accommodate participants working during the week. The FGDs were held in private areas by a trained research assistant with experience in conducting qualitative research. Furthermore, open-ended questions guided the discussions (Box 1), which were audio-recorded with permission from the participants. To minimise bias, all follow-up questions were also framed in an open-ended manner.

BOX 1 Exploratory question and probes.

What is your opinion about the use of family planning?

Probes:

1. What advantages do you see in the use of family planning?
2. What disadvantages or problems do you feel are there with the use of family planning?
3. How would you feel if your wife or partner used family planning?

The audio-recordings were later transcribed verbatim in Xitsonga by a research assistant. Subsequently, the transcribed data and the audio-recordings were cross-checked by the principal researcher and another Tsonga-speaking research assistant. Finally, the typed, de-identified narratives were translated into English by a language expert. Thematic analysis was conducted using a deductive coding process. This involved checking the transcripts several times and implementing colour coding. From these codes, patterns were identified and themes were generated. The thematic analysis was done by the principal researcher and assistant researcher independently, and the findings were compared thereafter. Themes were also reviewed against the data set to ensure their usefulness and accuracy. In addition, the findings were checked by an independent researcher who is a public health specialist.

During the abovementioned study procedures, trustworthiness was enhanced by ensuring *credibility* through triangulation, peer review and member checking. By providing detailed descriptions of the participants, as well as the setting of the research, the *transferability* of the conclusions of this study to other similar settings has been established. *Dependability* and *confirmability* was assured by describing the step-by-step audit trail, as well as triangulation. *Confirmability* was further bolstered by peer review.

### Ethical considerations

Ethical approval was obtained from the Turfloop Research and Ethics Committee (project number TREC/33/2018) with permission from the Limpopo Provincial Department of Health Research Committee and Department of Health Mopani District. Written consent was received from all the participants after they had been informed verbally and in writing of the research details. To ensure confidentiality, FGDs were held privately and all data were de-identified. Furthermore, no other personal information, including occupation, was included in the report.

## Results

Of the 26 men included in the FGDs, eight (31%) were aged 30 years or younger, five (19%) were between the ages 31 and 45 and the remaining 13 (50%) were over 45 years of age. Most participants were married or cohabiting and had some secondary education.

**FIGURE 1:**
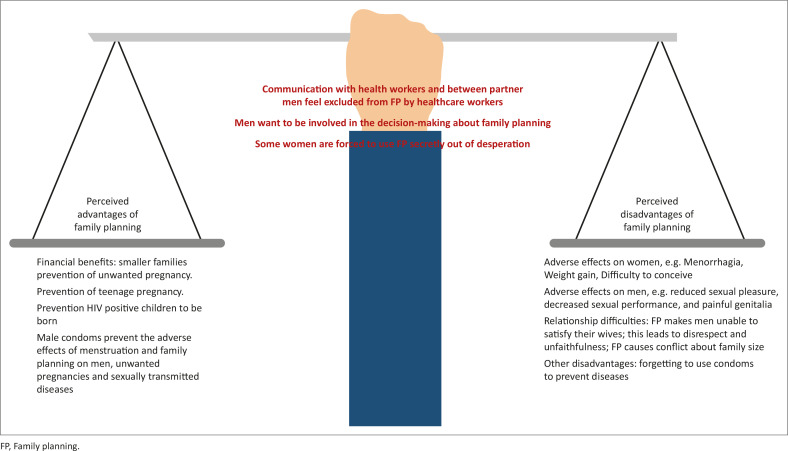
Diagrammatic depiction of the three major themes and subthemes: a balanced scale with the perceived advantages of family planning (FP) on the one side and the perceived disadvantages of FP on the other. The third major theme is communication with men about FP, which is depicted as the central arm of the scale.

Three major themes emerged from the research findings (Figure 1), namely the perceived advantages of FP, the perceived disadvantages of FP and communication with men about FP. The perceived advantages included several subthemes such as the financial benefits of having smaller families, preventing unwanted pregnancy, teenage pregnancy and not giving birth to HIV-positive children. Furthermore, using male condoms was seen to prevent the adverse effects of menstruation and FP on men. Additionally, male condoms also prevent unwanted pregnancies and sexually transmitted infections (STIs).

The subthemes that emerged under perceived disadvantages included weight gain in women, menorrhagia and difficulty conceiving. Adverse effects on men included decreased energy, decreased sexual performance and painful genitalia. Family planning use was also associated with marital difficulties, since the adverse effects of FP made men feel that they had failed to satisfy their female partners, resulting in disrespectful or unfaithful women. Additionally, hormonal FP was believed to make the youth forget to use condoms to prevent disease.

The third major theme acts as a link between the first two themes, and this is communication. Men feel excluded by healthcare workers from FP discussions. They want healthcare workers to educate them about different FP methods, the mechanisms of action and the risks. Men were also against the secretive use of FP methods by their partners, and they felt that they should be included in decision-making regarding FP. However, they did acknowledge that some women were forced to use FP because of financial constraints. The quotations supporting the major themes and sub-themes are presented in Box 2.

BOX 2 Major themes, subthemes and supporting quotations.

**Table:** 

**Major theme 1**	**Perceived advantages of family planning (FP)**
Financial benefits: Smaller families	‘I have three children, and I am okay with these three, as I can see that the rate of unemployment is too high. I do not want to end up stealing, so I think contraceptives are good.’ (59 years old, married, primary education)
Prevention of teenage pregnancies	‘I think it can also help, especially when you have children that are still at school, of which if they use contraceptives even if they go out on a drinking spree and or happen to be raped, they will be protected from falling pregnant as well as diseases.’ (48 years, married, secondary education)
Preventing birth of HIV-positive children	‘There are many diseases; people should first go for a check-up to avoid giving birth to a sick child. People must first consult the doctor and get checked, and when they are given a clean bill of health, they can have a baby. This is done to avoid a situation where you find that a child has been born sick and must take prescribed medication to control the disease. It is not necessary.’ (46 years, married, secondary education)
Male condoms prevent the adverse effects of menstruation and FP on men, unwanted pregnancies and STIs	‘A condom protects, and it is good because if she is on her periods, I use a condom. Also, when she tells me that she was injected yesterday, it helps.’ (49 years old, married, secondary education) ‘It is a good thing to use protection. A condom protects from both falling pregnant as well as contracting diseases.’ (29 years, cohabiting, secondary education)
**Theme 2**	**Perceived disadvantages of FP**
Adverse effects on women: menorrhagia	‘Family planning is a good thing, but women differ; some experience longer periods that end up disturbing their sex life and therefore drives me as a partner to cheat.’ (49 years, married, secondary education)
Weight gain	‘At times, they gain weight with swollen legs that are so huge to the extent that shoes cannot fit, and it is so scary, but despite all this, she continues with the injection. When you have sex with her, you do not enjoy it.’ (46 years, married, secondary education)
Difficulty conceiving	‘… [*I*]t [*injection*] stays in the body for longer, making it difficult for a woman to conceive.’ (35 years, cohabiting, secondary education)
Adverse effects on men: reduced sexual pleasure and decreased sexual performance	‘It is no longer possible to have many rounds of sexual intercourse with a woman. We are unable to do it because of the injection.’ (30 years old, cohabiting, tertiary education)
Male condoms reduce sexual pleasure	‘I hate using condoms. There is a difference when you use condoms. It feels different when I use a condom as opposed to when I am without, i.e. sex is not the same.’ (46 years, married, secondary education)
Painful genitalia	‘When contraceptives are used, it creates problems for a man, such as draining his energy. She skips one or three months without menstruating, and you then have sex with her; you absorb that dirt. … [*Y*]ou will also skip three months. I will know that I have absorbed that dirt when I urinate, then my urine comes out dark at first, then clears as it goes. It affects the kidneys. The balls [*testes*] become affected too. I once met a woman who told me that she had skipped her periods for two to three months, and she is using an injection, so we had sex, and I felt a burning sensation on my penis. I then realised that it is because of the injection.’ (60 years, married, primary education).
Relationship difficulties: women think their partners are uninterested in them	‘She then thinks that I do not want to be intimate with her, yet I want to be intimate, but I am weak, and she ends up thinking that maybe I am cheating on her and then goes out and cheat.’ (60 years, married, primary education)
Women become unfaithful	‘I go home so that I can always be with her because since she is using contraceptives, she can easily have an affair.’ (29 years, secondary education)
Women become disrespectful	‘Because of poor erection and loss of energy, we are disrespected by our spouses, and our families are destroyed.’ (52 years, married, secondary education)
Sexual boredom	‘What my brother is explaining here is true. Because you have sex with her every day, she ends up not having feelings for you, and as a man, I will end up cheating on her. In the older days, a man could stay in Johannesburg until the baby starts crawling, and it was not a problem.’ (60 years, married, primary education)
Conflict about family size	‘In our families, we have wives, and our wives no longer want to have children. In most instances, once a woman gives birth to a child, she usually says that this one child is enough despite the husband’s wishes of having more children. I am even considering divorce.’ (55 years, married, primary education)
Other disadvantages of FP: forget condoms to prevent diseases	‘We forget about diseases; we tend to forget that contraceptives do not prevent diseases, and once they [*youth*] start using them, they forget to use condoms.’ (30 years old, cohabiting relationship, secondary education)
**Theme 3**	**Communication with men about FP**
Men feel excluded from family planning by healthcare workers	‘Some [*women*] do not inform their husbands when they go for family planning; things are not done well in the clinics. At the clinic we are told to excuse ourselves when it is time for family planning education. Both of us must be able to understand. They must include us because we are also parents, the mother is not the only parent responsible for the child but both parents. All of us must understand that family planning is not meant to destroy a family.’ (30 years, relationship, tertiary education)
Communication between partners about family planning	‘Let us say you are keeping a secret from me; I will be angry because it is like you have taken out an engine and all my efforts are in vain. But if she suggest to me that she is thinking of taking out the engine and we both agree to do it, I will be very hurt if she does it alone.’ (30 years, cohabiting, secondary education)
	‘… [*W*]ith me, the contract [*marriage*] is over … if I found out that she has been using contraceptives secretly.’ (53 years, married, some secondary education)
Some women are forced to use family planning secretly out of desperation	‘There are instances where a woman decides to use contraceptives because I have impregnated her and she has a child, but I am failing to take care of the child [*financially*], and life is difficult, so she then decides to use contraceptives secretly.’ (29 years, cohabiting, secondary education)

STI, sexually transmitted infection.

Taken together, it is clear that men are ambivalent about the use of FP. There are perceived advantages and disadvantages. Communication has the power to tip the balance of the scale in favour of FP when men are included in FP education and decision-making.

## Discussion

Men are aware of FP and its benefits. Nettey et al.^7^ found that most participants agreed that children born into smaller families were more likely to succeed in life. Socio-economic reasons are significant drivers in men’s interest in FP.^2,3,8,9^ Consequently, efforts to improve male involvement in FP should include information on how it may contribute to greater financial security.^4^

Nettey et al.^7^ also reported that men felt condoms prevented unplanned pregnancy and Sexually Transmitted Diseases, specifically when having sex with women they did not trust. Our findings suggest that men also use condoms when their spouses are menstruating or a day after receiving an injectable FP method to protect themselves from the perceived adverse effects of FP. This is a finding that has not yet been described in the literature.

The perceived physical adverse effects for women reported in this study were, namely, menorrhagia, weight gain and difficulty conceiving. This finding is consistent with other studies that found that weight gain and menorrhagia were reported by men as side effects experienced by their partners.^4,5,6^ However, difficulty conceiving has been ascribed to hearsay, and fear of adverse effects is often rooted in an overestimation of rare complications or based on unvalidated rumours.^6,7^ Some of our participants also reported that when women gain weight, they become sexually unattractive. Menorrhagia and weight gain were thus seen as precursors to infidelity in men. In the literature, fear of perceived side effects or adverse health beliefs have been described as a barrier to men’s involvement FP.^4^

Even though participants were aware that condom use can protect against the risk of pregnancy and STIs, their widespread view was that condoms lower sexual pleasure. This perception about condom use among men may negatively affect their adherence to safe sex practices. In the present study, men reported that they especially used condoms when their female partner was menstruating or had received injectable contraceptives or when having sex with a female partner they did not trust. This finding is corroborated by other similar studies.^10,11,12,13^

Some of our participants believed that hormonal FP causes an accumulation of ‘dirty blood’ in the woman’s body, which in turn triggers several symptoms in men, including weakness, decreased sexual pleasure, decreased sexual performance and painful genitalia. This indicates a lack of information on the mechanism of action of hormonal FP methods in preventing pregnancy.

Men in this study considered FP use to potentially contribute to conflicts in relationships. This was linked to a perception that men who suffer the adverse effects of FP may fail to satisfy their women sexually, resulting in disrespectful and unfaithful women. Several studies have reported similar views, where men believe FP makes women unfaithful because they are not worried about pregnancy.^4,10,11^ Men are often resistant to approve contraceptive use, primarily out of fear of indirectly encouraging their female partners to be unfaithful.^9^ Men’s mistrust of their female partners is a significant contribution to the association of FP with promiscuity.^13^

Furthermore, some participants felt that FP caused conflict because of a disagreement about the number of children they wanted compared to their partners. These findings are consistent with other studies that show how most cultural beliefs and practices in African countries place a high value on large families.^4,9,10^ In some instances, where women are keen on using FP, it leads to women being subjected to physical abuse and battering.^9,10^

The fact that men raised these concerns demonstrates the importance of engaging men in addressing the low FP uptake in South Africa. The opposition by the male partner has been identified as a significant factor contributing to FP discontinuation tendencies in the country.^10^

Men strongly disapprove of unilateral decisions regarding contraceptive use by their female partners,^8,9^ although some see FP as a woman’s domain.^4,14,15^ However, the secretive use of contraception is practised by women to acquire some degree of sexual autonomy in the context of restrictive gender norms.^9^ Communication is vital, and in the absence of honest communication, women often incorrectly perceive their partners to be opposed to contraceptives, resulting in the covert use of contraception or rejecting it altogether.^16^ Including male partners in decision-making will not only improve communication between partners but also enable men to support their female partners and share the responsibility of using contraceptives consistently and continuously.^10^ Several studies have found that FP services that mainly target women result in men feeling ignored, and this is a factor that limits male involvement in FP.^8,9,17^

In South Africa, most men are still patriarchal, and the suggestion that they want to be better informed about all FP methods is thus very encouraging. This underscores the need to educate men about the health and socio-economic benefits of FP, while explaining the possible side effects and dispelling any myths. Increased male awareness about the value of FP for both child spacing and limiting birth is invaluable, and to achieve this communication is the key.

### Limitations of the study

Although attempts were made to reduce bias and errors, they could still have been introduced in several ways. Some participants might have chosen their responses to please the researcher, and others may have felt too embarrassed to share their opinions on FP. Translating the participants’ responses from Tsonga to English could additionally have given rise to inaccuracies. While the findings of this study are not transferable to any society, a description of the setting has been provided in an attempt to clarify in what context our results may be applicable.

## Conclusion

Results from this study have shown that men are aware of the advantages of FP and have favourable attitudes towards its use. However, men also have doubts about allowing their female partners to use FP because of certain perceived disadvantages, including several misconceptions. These comprised decreased sexual performance, decreased sexual pleasure and painful genitalia in men. In women, FP was seen to cause promiscuity and disrespect, as well as difficulty conceiving. These findings suggest a lack of adequate information about FP methods and their risks among men. Men are interested in learning more about FP; however, they are against the secretive use of FP by their female partners and have blamed healthcare service providers for excluding them in FP discussions.

### Recommendations

There is a need to increase male participation in FP decisions, at FP clinics, as well as at antenatal and postnatal clinics. This will help to improve men’s knowledge about the different FP methods and correct misconceptions, which will lead to enhanced FP acceptance and use. Healthcare workers must be encouraged and trained to include men in FP education and ultimately in FP decision-making. Community leaders, opinion leaders and the media should also be involved, as they can play an important role in advocating for the utilisation of modern FP methods.

## Data Availability

The principal author, N.D.R., has all data stored on her computer.
